# Diversity of Lipid Function in Atherogenesis: A Focus on Endothelial Mechanobiology

**DOI:** 10.3390/ijms222111545

**Published:** 2021-10-26

**Authors:** Stanislav Kotlyarov

**Affiliations:** Department of Nursing, Ryazan State Medical University, 390026 Ryazan, Russia; SKMR1@yandex.ru

**Keywords:** atherosclerosis, lipid metabolism, endothelium, mechanobiology, cholesterol, plasma membrane, blood flow

## Abstract

Atherosclerosis is one of the most important problems in modern medicine. Its high prevalence and social significance determine the need for a better understanding of the mechanisms of the disease’s development and progression. Lipid metabolism and its disorders are one of the key links in the pathogenesis of atherosclerosis. Lipids are involved in many processes, including those related to the mechanoreception of endothelial cells. The multifaceted role of lipids in endothelial mechanobiology and mechanisms of atherogenesis are discussed in this review. Endothelium is involved in ensuring adequate vascular hemodynamics, and changes in blood flow characteristics are detected by endothelial cells and affect their structure and function.

## 1. Introduction

Atherosclerosis is one of the key medical and social problems of the present age [1]. Diseases associated with atherosclerosis are among the most important causes of medical care, disability, and mortality, largely forming the epidemiology of so-called noncommunicable diseases (NCDs) [2,3,4]. The clinical manifestation of atherosclerosis is associated with the development of ischemic diseases due to progressive arterial damage. Ischemic heart disease, ischaemic stroke, and peripheral arterial disease of the lower extremities are characterized by a wide prevalence and carry a heavy economic and social burden [5,6]. The economic costs of atherosclerosis are a serious burden to patients and their families as a consequence of the health care systems of many countries, making it necessary to study and understand all aspects of atherogenesis better [1,3,7].

The complexity of the problem of atherosclerosis is also due to the fact that atherogenesis begins long before the clinical manifestation [8]. At the same time, the arsenal of therapeutic remedies for clinically advanced stages is currently limited and does not allow successful treatment for all patients.

Atherosclerosis is common mainly in older age groups with risk factors such as obesity, hypodynamia, hypercholesterolemia, smoking, arterial hypertension, and diabetes mellitus [9]. Correction of these risk factors can reduce the likelihood of developing atherosclerosis or reduce the rate of its progression. Numerous epidemiological and experimental studies have improved our understanding of the relationship between lipid metabolism disorders and atherosclerosis. The classical experimental works of N.N. Anichkov were one of the first confirmations of the role of impaired lipid metabolism in the mechanisms of atherogenesis [10,11]. Today, the main therapeutic efforts in the prevention and treatment of atherosclerosis and related complications are focused on the correction of impaired lipid metabolism [12,13].

Despite the systemic nature of the key risk factors and long-term progressive development, the lesion of the arteries is not diffuse. Some areas are most susceptible to the development of atherosclerotic lesion, including the coronary arteries, bifurcations of the carotid arteries, and branches of the arteries of the lower extremities (i.e., those areas of the arteries where there are bends and branches [14,15,16]). These observations led to the conclusion that local hemodynamic factors that affect the vascular wall and, in particular, the endothelium, are responsible for the heterogeneity of the distribution of atherosclerotic lesions [14,17,18,19].

There is increasing evidence that the formation of atherosclerotic plaques begins with damage to the endothelium, which contributes to the adhesion of circulating immune cells that trigger further progression of atherosclerosis [20,21,22]. The development of high-precision research methods made it possible to determine the leading role of the endothelium in vascular function. The endothelium is considered not just as a mechanical barrier, but a key participant in vascular biology [21,22,23]. It is actively involved in atherogenesis, demonstrating both increased permeability to plasma molecules, and increased adhesion and intraparietal migration of monocytes. At the same time, the endothelial cells themselves are characterized by altered metabolism, inflammatory activation and structural modification [22,24,25,26]. The endothelium is involved in the production of many biologically active factors associated with inflammation, which is considered to be one of the most important links in atherogenesis [27]. However, the initiating factors and the initial links in the complex chain of processes leading to endothelial function impairment and atherosclerosis remain largely unknown.

This review focuses on the participation of lipids in the complex dynamic interaction between biophysical and molecular processes in the plasma membrane of endothelial cells, which are related to endothelial mechanobiology, endothelial cell function and impairment of all these processes in atherosclerosis.

## 2. Current Understanding of Endothelial Function

Endothelial cells have a unique biology related to their location and function. They form a single cellular monolayer that lines all blood vessels, from the heart to the capillaries [23]. Located at the interface between blood and surrounding tissues, endothelial cells control the transport of various substances and cells from the bloodstream to the tissues and back. In addition to regulating permeability, the endothelium participates in providing anticoagulant properties of blood, controlling the diameter of the lumen of blood vessels [28,29].

Regulation of vascular lumen is a key hemodynamic function of endothelial cells, which is provided by the production of nitric oxide, prostacyclin, and endothelin [29,30]. Disorders in the formation or bioavailability of nitric oxide of endothelial origin and associated adverse changes in vascular reactivity in cardiovascular medicine are usually referred to as “endothelial dysfunction” [20]. This term and the term “endothelial cell dysfunction” are used to describe other changes in the functional phenotype of endothelial cells, including those associated with atherosclerosis [20,31,32].

An important conceptual achievement of vascular biology, which expanded the boundaries of our understanding of atherosclerosis, has been the demonstration that the endothelium can regulate the behavior and function of other cell types both in the vessel wall and in the circulating blood, producing many different biologically active factors [20,33,34].

In this context, it is necessary to note the concept of endothelial activation and its significance in the pathogenesis of atherosclerosis [35,36]. The current paradigm of endothelial activation involves biochemical (including cytokines such as tumor necrosis factor alpha (TNF-α) and interleukin-6 (IL-6), growth factors, bacterial endotoxins and other stimuli) and biomechanical (related to the hemodynamic characteristics of blood flow) cell stimulation [37,38,39]. Endothelial cell activation involves a stimulation stage (type I activation) which includes the participation of existing proteins in the cell and an activation stage (type II activation) representing a delayed response involving de novo protein synthesis [40]. The genes whose expression is associated with type II endothelial cell activation encode cytokines, chemokines, adhesion molecules, and coagulation-related factors [40]. Endothelial cell activation is thus determined by the endothelial expression of cell adhesion molecules such as vascular cell adhesion molecule 1 (VCAM-1), intercellular adhesion molecule-1 (ICAM-1) and E-selectin and is a crucial step in the process of monocyte migration into the vascular wall. Interleukin-8 (IL-8), the synthesis and secretion of which is observed when proinflammatory cytokines affect endothelial cells, can also be considered as an activation marker [40].

According to this concept, endothelial cell activation and endothelial dysfunction are not synonymous, but rather characterize morphological and biosynthetic processes reflecting stages of change in functional cell activity since exposure to a damaging factor [41]. Interestingly, NO can limit endothelial activation and inhibit monocyte adhesion [39,42]. These findings reinforce the role of nitric oxide as a node providing a link between hypercholesterolemia, smoking, turbulent blood flow, and reduced NO production and endothelial activation [43].

Thus, endothelial activation plays an important role in monocyte adhesion and is a key step in the initiation and progression of atherosclerosis [42].

Since the hemodynamic conditions in different parts of the cardiovascular system differ, endothelial cells, in accordance with their localization, demonstrate a certain structural and functional heterogeneity [44,45,46].

New keys to understanding the function of the endothelium came from the analysis of its evolution. Only vertebrates, which constitute about 3–5% of the total biodiversity of living creatures, have endothelial lining of blood vessels. The development of the endothelium is assumed to have occurred in the ancestral vertebrate 540–510 million years ago [47]. Thus, a significant number of invertebrates have no endothelium, and their blood vessels are lined with an extracellular matrix [47,48,49]. At the same time, the blood vessels of some invertebrates, including cephalopods, annelids and amphioxus are covered with cells [47,48,50,51,52,53]. These cells (amoebocytes) do not form dense intercellular connections and are not connected to the basal lamina. However, they can provide closed blood circulation and maintain a sufficiently high blood pressure [49]. Amoebocytes are a type of circulating hemocyte and are supposed to be an evolutionary precursor of endothelial cells [47]. Interestingly, human endothelial cells have some functions of innate immunity that macrophages perform, including the function of phagocytosis, cytokine secretion, antigen presentation, detection of pathogen-associated molecular patterns (PAMPs) and damage-associated molecular patterns (DAMPs), and also have the ability to migrate [54,55,56]. In addition, thrombus leukocytes express more endothelial cell-specific angiogenic markers than peripheral blood leukocytes in patients with acute coronary syndrome, which suggests the possibility of trans-differentiation [57]. Given the close association of endothelial cells with the immune response [56], there is also an assumption that endothelial development is associated with the development of adaptive immunity in vertebrates.

It is assumed that the presence of an endothelial monolayer allowed vertebrates to provide the possibility of increasing arterial blood pressure by reducing the loss of plasma and formed elements through tight contacts of endothelial cells, as well as to reduce the volume of circulating blood with better perfusion capabilities through an extensive network of capillaries [47,58].

Thus, the maintenance of hemodynamic characteristics of blood flow, the regulation of the density of intercellular connections and vascular wall permeability are closely related to endothelial function. In areas prone to atherosclerosis that have impaired hemodynamic characteristics of blood flow, endothelial cells have structural and functional changes, and also demonstrate increased permeability to macromolecules, increased proliferation and apoptosis, and increased adhesion of blood monocytes [24,25].

## 3. Hydrodynamic Characteristics of Blood Flow

Arterial endothelial cells are in complex hemodynamic conditions, constantly exposed to several physical forces due to the presence of blood pressure and the pulsating nature of blood flow [59].

**Figure 1:**
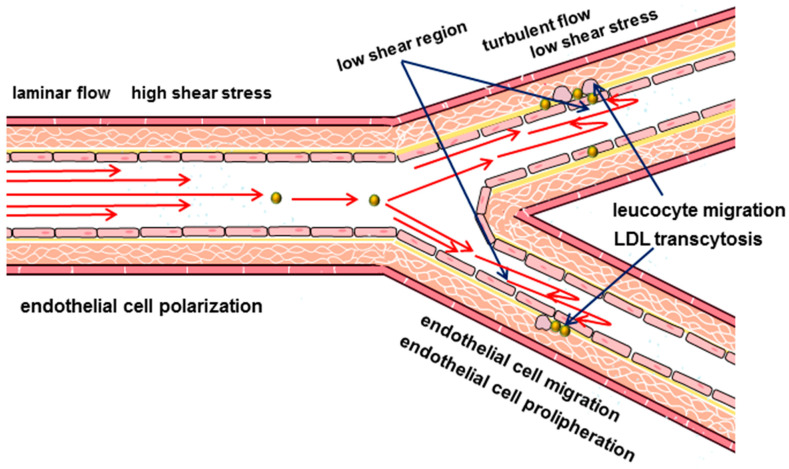
Scheme of hemodynamics in arterial bifurcation. Regions with low shear stress are associated with the development of atherosclerosis.

According to modern concepts, peripheral vascular blood flow is considered as laminar. This physical property implies the movement of blood in ordered parallel layers along the long axis of the artery without mixing these layers. And the velocity of the layers varies from the minimum along the vascular wall, gradually accelerating closer to the center of the vessel. The friction force that acts on the endothelial cells by the boundary blood flow is called the shear stress. Shear stress is considered one of the key hemodynamic determinants of endothelial function [60]. The shear stress acts parallel to the surface of the endothelial cells and depends on the flow rate and viscosity of the blood, which in turn can also vary [60,61,62]. The magnitude of the shear stress in the arteries is higher than in the veins and varies from 10 dyn/cm^2^ to 40 dyn/cm^2^, while in the veins it is approximately from 1 dyn/cm^2^ to 6 dyn/cm^2^ [63,64,65,66]. In the arteries, the shear stress depends on the phase of the cardiac cycle, increasing during systole and decreasing in diastole [67,68]. The geometry of the vessels, such as bends and bifurcations, also affects the nature and values of the shear stress [24,62,69]. In the straight parts of the arterial tree when blood flow is laminar, shear stress is high and directional. While laminar blood flow is considered physiological, the appearance of turbulence is seen as an important initiating factor for atherosclerosis [70]. The transition to turbulence is associated with curvature, stenosis, branching of the arteries and is accompanied by the formation of disordered chaotic flows (Figure 1). Such impaired blood flow patterns are characterized by an irregular distribution of small shear stresses [24,67,71].

Obviously, this interpretation of hemodynamics is simplified, since the complex geometry of many vessels does not always predispose to laminar blood flow [72]. And the very characteristic of turbulence in vascular hemodynamics is the subject of discussion [70]. It is shown, for example, that the hemodynamic characteristics of some main vessels imply turbulent blood flow, which ensures uniform mixing of cells, which would be impossible under laminar flow conditions [72]. Besides, in addition to the shear stress, the arteries are constantly exposed to blood pressure influence, which act perpendicular to the vascular wall [28,73,74,75]. There is evidence of the influence of other physical forces on endothelial cells, including those related to blood circulation, as well as interaction with the extracellular matrix [28,69,76]. Thus, the existing models today cannot fully take into account the whole variety of hemodynamic conditions in which the endothelium is located.

As it has been noted earlier, the endothelium is not a passive participant, but is actively involved in hemodynamic regulation responding to changes in blood flow [77]. In response to an increase in blood flow velocity, which corresponds to an increase in shear stress above a certain level, the endothelium dose-dependently increases nitric oxide production [78]. Nitric oxide causes relaxation of vascular smooth muscle cells, thereby providing vasodilation and reducing the blood flow velocity and, accordingly, the shear stress to the necessary optimal values [79,80,81,82].

In addition, high shear stress induces dose-dependent expression of Krüppel-like Factor 2 (KLF2) transcription factor, which provides anti-inflammatory function of endothelial cells [60]. In addition, KLF2 is important for the alignment of endothelial cells under the influence of shear stress, which is part of the mechanism of cell adaptation to hemodynamic conditions [83].

The ability of endothelial cells to respond to changes in blood flow characteristics actively allows them to adapt most effectively to the action of hemodynamic forces. Changes in blood flow characteristics can affect the orientation and morphology of endothelial cells [28,84,85,86,87,88,89,90,91,92,93]. Under conditions of laminar blood flow with a relatively high shear stress (20 dyn/cm^2^) endothelial cells become elongated and orient themselves in accordance with the flow direction [28,90,94,95,96]. Moreover, the elongation and polarization of endothelial cells correlate with the magnitude of the shear stress [62,77,97] (i.e., in the arteries the polarization of endothelial cells is more pronounced than in the veins [98]). The polarization of endothelial cells includes an asymmetric organization of cellular organelles, in which the Golgi apparatus is located in front of the nucleus with respect to the flow direction [99,100,101,102,103]. The movement of the nucleus is associated both with its mechanical displacement under the action of hydrodynamic resistance, and with the action of the actomyosin cytoskeleton and microtubules [98,104,105,106,107]. When exposed to turbulent or eddy flows that do not have a predictable direction and have low shear stress values, the alignment of endothelial cells does not occur [28,108], the cells have a rounded shape and a disorganized orientation of the Golgi apparatus [101,109]. The flow-induced polarization of endothelial cells is a dynamic and reversible process. It has been found in experiments, that the majority of endothelial cells are polarized within 4.5 h after the start of intensive blood flow [110]. The polarization and orientation of the endothelium in the direction of blood flow is an important mechanism, since it allows the most effective optimization of the flow-induced effect on the cell surface and intercellular connections [111,112,113,114,115,116]. Conversely, cells that do not have a preferred orientation in conditions of impaired blood flow are assumed to be unable to control fully the permeability through intercellular junctions for atherogenic lipids.

Another atheroprotective mechanism of stable laminar flow is its ability to inhibit endothelial cell proliferation [117,118]. In contrast, disturbance of stable laminar flow stimulates cell proliferation and may contribute to impaired endothelial monolayer stability and increased intercellular permeability [117,118].

Another mechanism that provides effective protection of the endothelial monolayer during steady laminar flow is the regulation of endothelial cell migration rate. A high shear stress can induce endothelial cell migration, whereas a disturbed flow does not have such an effect [77,119].

The ability of endothelial cells to modify their shape and orientation is associated with the reorganization of their cytoskeleton, which is rebuilt in accordance with the blood flow [28,69,77,94,120,121,122,123,124]. Endothelial cells aligned in the direction of the laminar flow with a high shear stress have well-organized long parallel actin fibers in the central part of the cell [24,93,94,95,125,126,127,128,129], while a low shear stress contributes to the peripheral redistribution of actin microfilaments [28,121,126,128,130,131,132,133,134]. These data confirm the participation of the cytoskeleton as the main mechanism for the transmission of hemodynamic forces in endothelial cells, in which actin filaments are of particular interest, taking into account their connection with transmembrane integrins [135].

Thus, endothelial cells are constantly exposed to several physical forces with different vectors and are able to detect them and convert them into intracellular signals that affect cellular functions [136]. The current concept of vascular hemodynamics suggests that laminar blood flow contributes to the polarity of endothelial cells, which is characterized by an elongated cell shape oriented in accordance with the direction of blood flow and an asymmetric arrangement of cellular organelles, such as the nucleus and Golgi apparatus. This cell polarity is considered anti-inflammatory, as opposed to pro-inflammatory in turbulent flow [101]. It should be noted that although the concept of low and/or oscillatory shear stress as an initiating atherogenic mechanism is predominant, it does not provide answers to all questions and is still an object of discussion [137].

Shear stress magnitude and the rate of its change are the key hemodynamic characteristics that affect the mechanobiology of the endothelium [138]. The plasma membrane and its connection with the actin cytoskeleton of the endothelial cell are part of the mechanism of mechanical transduction [139,140]. Taking into account the fact that the interaction of hemodynamic forces with the cell begins with the plasma membrane, the mechanisms of transformation of cellular deformation into molecular signaling pathways mediating mechanical transduction are of great importance [139,140,141].

## 4. Lipids in Endothelial Mechanobiology

The results of numerous studies have improved and systematized our understanding of the structure and function of plasma cell membranes [142,143]. The plasma membrane is a complexly regulated multicomponent system that not only performs the function of separating a living cell from the surrounding space, but also organizes various processes that provide many cell functions.

Cholesterol is an important component of plasma membranes. Cholesterol content in the plasma membrane is complexly regulated and is provided by many factors including biosynthetic and transport processes. Due to its chemical structure, cholesterol participates in the lateral organization of the lipid bilayer of the plasma membrane, which largely determines its properties and functions [144]. The chemical structure of the cholesterol molecule determines its location in the plasma membrane [145]. The hydroxyl group of cholesterol is located near the lipid-water interface between the polar head groups of phospholipids, while the polycyclic sterane ring is located in the thickness of the membrane [146]. The rigid sterane ring of cholesterol has an asymmetric structure, including a flat α surface and a β surface with aliphatic groups. Sphingolipids usually interact with the α-surface of cholesterol, and transmembrane domains of proteins interact with the β-surface [147,148]. Due to this, the polycyclic sterane ring of cholesterol provides a denser packing of lipids, thereby increasing the viscosity of the lipid bilayer of the membrane.

The cholesterol content has a significant effect on the main mechanical parameters of the plasma membrane, determines its stiffness, elasticity and resistance to rupture under load [149,150,151,152,153,154]. The plasma membrane fluidity depends on cholesterol content, and cholesterol can affect the fluidity of the membrane in different ways at its different depths [146,155,156,157]. It was found out that in the presence of cholesterol, the membrane fluidity decreases near the surface of the bilayer and increases near the center of the bilayer, since the rigid sterane ring of cholesterol reaches only a depth approximately equal to the position of the C9–C10 of carbon atom in acyl chains [146]. This fact may be important for ensuring the optimal spatial arrangement of membrane proteins.

Cholesterol is unevenly distributed in the plasma membrane, providing a unique lateral organization of most plasma membranes, which includes the simultaneous existence of a lipid ordered phase and a lipid disordered phase [146]. The lipid ordered phase is associated with cholesterol-rich microdomains of membranes, the so-called lipid rafts [158].

Lipid microdomains are special structures of plasma membranes that are enriched with cholesterol and sphingolipids and are signaling platforms that provide many important cellular functions. There are two types of lipid domains of the plasma membrane that differ in structure-planar lipid rafts and so-called caveolae, which are microdomains with a diameter of 70–100 nm invaginated into the cell. Caveolae may exist both in the form of single invaginations and in the form of rosette-like clusters [159,160,161]. The plasma membranes of endothelial cells contain a large number of caveolae, which suggests their important role in the specialized functions of these cells. Endothelial cells in the vessels of various organs contain different amounts of caveolae, which reflects their functional heterogeneity. It should be noted that caveolae are characteristic not only for endothelial cells, but also for other cells involved in atherogenesis (which, however, is beyond the scope of this review).

Caveolae are involved in several endothelial cell functions, including transcytosis of large and small molecules [160,162,163,164,165], and also act as platforms on which numerous signaling molecules are located [166,167,168]. One of the most well-known functions includes participation in the regulation of endothelial NO-synthase (eNOS) [160,169,170,171].

The structure of caveolae is supported by several proteins important for their formation and stabilization. Among the most significant are the structural protein caveolin-1 and the adapter protein cavin-1 [172,173,174,175,176]. Their expression is closely interrelated [172,177,178,179,180,181]. Caveolin-1 is key to caveolae formation as its loss results in the absence of caveolae, and conversely, expression of caveolin-1 in cells lacking caveolae causes their formation [181,182]. Interestingly, despite the importance of caveolae, animal models of *Cav1^−/−^* are viable, but have a number of cardiovascular defects and a shortened life expectancy [183,184]. Cavin-1 is necessary for the stabilization of caveolin-1 and its attachment to the cytoskeleton. In addition to its structural role, caveolin-1 is an important regulator of several molecular processes [180,185,186,187]. Through direct interaction with eNOS, caveolin-1 affects the activity of the enzyme negatively, which limits the production of NO [172,185,188,189]. Disruption of caveolae structure affects the activation of eNOS and vascular reactivity [168,190].

Experimental data indicate the multifaceted role of caveolin-1 in atherogenesis. It should be taken into account that caveolae are also present in other cells besides endothelial cells, for example, in cells of the myeloid line, which are also actively involved in atherogenesis [168]. The contradictory evidence for the function of caveolin-1 in the pathogenesis of atherosclerosis is that although caveolin-1 expression decreased in cells in the area of the atherosclerotic lesion during its progression [191,192,193,194,195], caveolin-1 deficiency reduces the development of atherosclerosis despite an increase in plasma lipid levels [196,197,198]. This dual effect may be due to the fact that caveolae are involved in the transendothelial migration of low density lipoprotein (LDL) [191].

In addition to their role in the organization of molecular processes, caveolae participate in providing some mechanical functions. They are considered as a reserve of the plasma membrane surface, which allows endothelial cells to undergo rapid changes in the cell surface area [199]. Thus, the flattening of caveolae is considered as a mechanism for rapidly increasing the surface area of cells, which prevents cell membrane damage when exposed to some physical factors [200]. An increase in arterial pressure has been shown to reduce the number of caveolae, resulting in the transformation of arterial pressure into an inflammatory vascular response [201]. Indeed, in *Cav1^−/−^* mice increased cardiac output leads to damage to the endothelial membranes, which confirms the participation of caveolae in the protection of plasma membranes from mechanical stimuli [202,203].

Experimental data indicate that in response to changes in the shear stress in the plasma membranes of endothelial cells, the ordering of the lipid organization changes, which affects some physical properties of the membrane, such as fluidity and viscosity [204,205,206,207]. The shear stress at laminar flow (10 din/cm^2^) leads to a rapid decrease in the lipid order of the plasma membrane, with the most pronounced changes in the ordered phases, as a result of which the caveolae also pass into a liquid disordered state [208]. The decrease in the lipid order depends on the intensity of the shear stress and is reversible [209]. This mechanism has a physical basis, since a similar decrease in the lipid order was observed in artificial membranes that were exposed to shear stress influence [208].

Shear stress can activate mitochondrial oxidative phosphorylation in endothelial cells through regulation of cholesterol content in plasma membranes [205].

It is assumed that the ordering of lipids in the plasma membrane varies depending on the nature of the physical action on the membrane, which allows endothelial cells to distinguish between shear stress and stretching [210]. Stretching is characterized by an increase in plasma membrane cholesterol content and a corresponding transition from a disordered lipid phase to an ordered lipid phase in some areas and a decrease in membrane fluidity [210]. On the contrary, cholesterol levels were decreased in response to shear stress, reducing lipid order and increasing membrane fluidity [210].

Lipid rafts are important platforms that provide a connection between the membrane and the cytoskeleton. It is shown that cholesterol plays a special role in the regulation of this relationship [150]. Cholesterol enrichment leads to decreased fluidity of plasma membranes [153]. However, in endothelial cells, the enrichment of the plasma membrane with cholesterol causes a decrease in its surface viscosity [150,211] and a weakening of the membrane-cytoskeleton adhesion [211,212,213,214]. Cholesterol depletion, although it leads to a disruption of lipid rafts, but instead of the expected decrease in the connection of the plasma membrane with the cytoskeleton, on the contrary, increases the stiffness of endothelial cells, which is due to increased attachment of the plasma membrane to the actin cytoskeleton [150,211,212].

Indeed, increased endothelial cell stiffness has been shown in atherosclerosis-prone areas with a disturbed flow structure, compared with areas of arteries characterized by higher shear stress values with a more uniform unidirectional blood flow [215].

Changes in the lipid structure of the plasma membrane also participate in the migration of endothelial cells. At the leading edge of the migrating cell, the microviscosity of the plasma membrane increases due to changes in cholesterol concentration. At the same time, caveolae and caveolin-1 show differences in the predominant localization in the migrating cell. Moreover, the nature of migration determines the peculiarities of the polarization of caveolae and caveolin-1 [216].

Information about another mechanosensory mechanism associated with the induction of neutral sphingomyelinase activity during changes in hemodynamic characteristics of blood flow is of interest. The activation of the enzyme occurs directly on the surface of endothelial cells, mainly in caveolae and leads to the formation of ceramides [217]. Ceramides are part of the mechanoreceptor pathway that detects external influences on the plasma membrane and leads to the activation of downstream mitogen-activated protein kinase [217,218,219]. Ceramides generated by sphingomyelinase can act as a signaling molecule for apoptosis. Sphingomyelinase can affect the physical properties of the membrane, converting sphingosine into ceramides [220,221,222]. Due to their biophysical properties, ceramide molecules self-associate due to hydrophobic interactions, creating microdomains with unique biophysical properties [220]. They are characterized by high structural rigidity, mechanical stability and compactness of lipid bilayers [221]. Ceramides may contribute to the development of endothelial dysfunction. It has been shown that the levels of ceramides are increased in plasma in patients with coronary artery disease [223].

In this regard, inhibition of acid sphingomyelinase is seen as a potential therapeutic target for impaired vascular function. Inhibition of acid sphingomyelinase and ceramide formation has been shown to improve endothelium-dependent vasodilation in diabetic animals [224]. Drugs belonging to the group of functional inhibitors of acid sphingomyelinase (FIASMA) are considered as promising agents for the treatment of many diseases associated with increased acid sphingomyelinase activity, but data on their role in atherogenesis are limited, highlighting the need for better study [225,226,227]. The complex role of sphingomyelinases in mechanotransduction is demonstrated by the fact that inhibition of neutral rather than acidic sphingomyelinase by scyphostatin results in impaired shear stress-induced mechanotransduction, demonstrating the involvement of neutral sphingomyelinase and ceramide formation as a primary and secondary mediator of downstream mechanosensory signal transduction. [219].

Thus, a change in the blood flow pattern affects not only the lipid organization of the membrane, but also its composition. It has been shown that the shear stress during laminar flow can control the endogenous metabolism of ether-containing lipids with an average alkyl chain length, which may be important in regulating the expression of VCAM-1 [218]. Experimental data indicate that the shear stress modulates VCAM-1 expression in response to TNF-α and dietary lipids through interferon regulatory factor-1 (IRF-1). At the same time, a low shear stress (2 din/cm (^2^)) causes an increase in the expression of VCAM-1 by 150%, and a high shear stress (12 din/cm(^2^)) leads to a 70% decrease compared to the static control [228]. In addition, laminar flow (shear stress 12 dyn/cm^2^, 12 h) increases the expression of stearoyl-CoA desaturase-1 (SCD1), an enzyme that performs the biosynthesis of monounsaturated fatty acids, which affect the fluidity of the endothelial cell membrane. The induction of SCD1 is mediated via a peroxisome proliferator-activated receptory (PPARy)-specific pathway and can act as an important mechanism for regulating plasma membrane fluidity [229].

Dyslipidemia is an important factor that, along with hemodynamic disorders, mediates the development and progression of atherosclerosis. High LDL levels are among the key factors of atherosclerosis. The cross-connections of dyslipidemia and hemodynamic characteristics of blood flow are of interest. It is known that the mechanical properties of the endothelium strongly depend on the effect of the oxidized form of low-density lipoproteins (oxLDL) on cells [213,230]. In a model of atherosclerosis in pigs receiving a diet high in fat and cholesterol, it was found that endothelial cells isolated from the aorta had significantly less deformation of the membranes, that indicated the fact that the cells were stiffer than cells isolated from the aorta of pigs receiving normal food. In addition to increasing endothelial stiffness under conditions of dyslipidemia, this study also demonstrated a significant increase in endothelial stiffness in response to oxidized modifications of oxLDL [215,230]. Experimental data have shown that influence of oxLDL on endothelial cells leads to the same effects on the biomechanical properties of the endothelium as cholesterol depletion [231]. These include disruption of lipid organization [232], increased cell stiffness [230,232], and increased sensitivity to shear stress [213,233]. These effects are thought to be mediated by the introduction of oxysterols into the plasma membrane [231].

oxLDL can disrupt the structure of caveolae, which leads to internalization and inhibition of eNOS activity mediated by the CD36 receptor [170,233,234,235,236,237]. In addition, oxLDL induces actin polymerization and the formation of F-actin stress fibers [238], which, in addition to increasing the stiffness of endothelial cells, is accompanied by an increase in the ability of endothelial cells to rearrange in the direction of flow [213]. The stiffness of cells associated with the hemodynamic characteristics of blood flow and oxLDL exposure increases their susceptibility to mechanical damage, which may contribute to increased atherogenesis [239].

In addition to the fact that oxLDL also induces ceramide production through hydrolysis of sphingomyelin [233,240,241,242]

## 5. The Intersection of Endothelial Biomechanical Properties and Protein Function

Plasma membranes perform many important cellular functions. Although membrane proteins play the main role in these processes, the results of studies over the past few decades have shown that lipids and lipid-protein interactions are important regulators of processes associated with plasma membranes [243,244,245]. Thus, the function of lipids has been significantly expanded from understanding their role as a structural component of the mechanical barrier separating the cell from the extracellular space surrounding it and being the basis in which functionally active proteins are located, to the structure that is involved in ensuring the function of these proteins [246,247]. Cholesterol can affect the function of some membrane proteins by participating in their spatial organization or through specific lipid-protein interactions [248]. Proteins that interact with cholesterol may contain certain amino acid sequences that are involved in this interaction. The amino acid cholesterol-binding domain (CRAC, cholesterol recognition/interaction amino acid consensus sequence), is one of the known sequences and has been identified in proteins that interact with or are regulated by cholesterol [147].

Since integral proteins are embedded in the lipid bilayer of the plasma membrane, a change in the biophysical properties of the membrane can change the conformation of proteins or their interactions. The membrane fluidity associated with lipid mobility is important for the interaction and functioning of membrane proteins. On the one hand, the lipid microenvironment should be sufficiently liquid to allow proteins to perform the conformational changes necessary for their function, but at the same time be sufficiently structured to provide them with adequate mechanical support [220,249,250,251].

**Figure 2:**
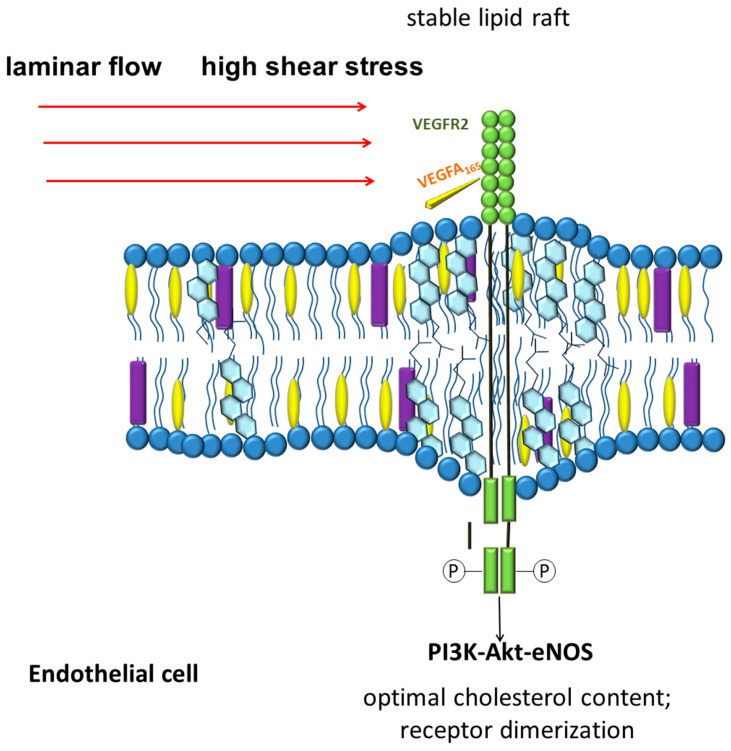
Schematic demonstrating the involvement of VEGFR2 in cross-linking blood flow characteristics and ligand-associated receptor activation. Stabilization of lipid rafts promotes receptor dimerization. Laminar blood flow promotes phosphorylation of VEGFR2.

The data on the participation of vascular endothelial growth factor receptor 2 (VEGFR2) as an important signaling pathway located at the intersection of chemo and mechanoreception connections are of great interest. VEGFR2 in endothelial cells is localized in lipid rafts [252]. The normal cholesterol content in the lipid rafts of endothelial cells stabilizes the dimeric state of VEGFR2 and affects its signaling pathway [252,253]. Disruption of lipid rafts results in impaired activation of the receptor signaling pathway in response to vascular endothelial growth factor (VEGF) exposure [252]. These and other data suggest the involvement of VEGFR2 as a sensor capable of simultaneously integrating chemical and mechanical signals (Figure 2) [254]. Laminar blood flow with a shear stress of 12 dyn/cm^2^ is able to rapidly activate VEGFR2 and its signaling pathways, including PI3K-Akt-eNOS, in a ligand-independent manner [255,256]. Interestingly, blood flow and VEGF-A can act together to influence endothelial cell alignment and polarity. When VEGFR2 is inhibited, the effect of shear stress and VEGF-A on endothelial cell alignment and polarity are lost, which allows to suggest the central role of VEGFR2 in these processes [254].

VEGFR2 is believed to be part of the mechanosensory complex of endothelial cells, which also includes vascular endothelial cadherin (VE-cadherin) and platelet endothelial cell adhesion molecule-1 (PECAM-1) [257]. Interestingly, the function of VE-cadherin, a key adhesion molecule in vascular endothelial cells responsible for maintaining endothelial barrier function, is associated with the cholesterol levels of plasma membranes. A decrease in cholesterol levels leads to a decrease in the function of VE-cadherin [258]. Interestingly, cholesterol crystals increase endothelial permeability with the involvement of VE-cadherin [259]. Subendothelial deposition of cholesterol crystals is associated with endothelial cell cholesterol overload with excess LDL uptake and is an important mechanism of lipid accumulation and endothelial cell dysfunction in atherogenesis [260].

G-protein-coupled receptors (GPCR) and their corresponding signaling molecules are mainly localized in lipid rafts, which are crucial for the transport and transmission of GPCR signals [261]. The lipid composition of the membranes has a strong influence on the conformational activity of GPCR [262]. The information that E-selectin and ICAM-1 bind to lipid rafts in endothelial cells after leukocyte adhesion is of interest [263]. The depletion of cholesterol from the plasma membrane disrupts the clustering of adhesion molecules and inhibits their association with src- kinases [263].

Thus, modulation of membrane lipid composition is a mechanism for regulating the activity of many membrane proteins that provide a number of important endothelial cell functions, including those related to the regulation of permeability. It should be noted that the maintenance of optimal membrane lipid composition is important, but so are the dynamics and direction of its change.

Moreover, lipids affect the function of proteins not only by providing conditions for their localization and optimal conformation, but also by posttranslational modification. Protein S-acylation (“palmitoylation”) plays an important role in the function of some endothelial cell proteins, such as caveolin-1, eNOS, PECAM-1 [264]. This posttranslational modification involves the addition of the C16 acyl chain to the cysteine residue in the protein by means of a thioester bond. This process is reversible, and is carried out by a complex of enzymes [265,266,267]. The main function of palmitoylation for many proteins is to increase the affinity for membranes, which contributes to its stable binding to lipid microdomains [265,268,269]. Palmitoylation of eNOS stabilizes its association with the membrane and localization in caveolae, and is necessary for its normal functional activity [264,270,271,272,273,274]. eNOS with impaired palmitoylation is characterized by lower nitric oxide production [176,266].

Taking into account the significant role of lipid balance in the function of membrane proteins, information about the participation of ATP binding cassette transporter A1 (ABCA1) and ATP binding cassette transporter G1 (ABCG1) in endothelial cell function is of interest. These members of a large family of ABC transporters participate in the formation of HDL, exporting mainly cholesterol from cells to extracellular acceptors. This process is called reverse cholesterol transport (RCT). Reverse cholesterol transport plays a significant role in atherogenesis, as it reduces the accumulation of cholesterol in macrophages, thereby having an anti-inflammatory effect [275]. However, the role of ABCA1 and ABCG1 in endothelial cells is less known than in macrophages [276]. Both transporters have been found to be involved in the regulation of some endothelial cell functions. They protect them from lipid overload with a high-cholesterol diet [277]. It has been shown that atherogenic stimuli, including hyperlipidemia, increase the expression of Abca1 in the endothelial cells of mice [278]. ABCA1 and ABCG1 are considered to be important participants in maintaining cellular cholesterol homeostasis and changes in their expression and functional activity are closely related to cholesterol content both in the whole cell and in the plasma membrane. In general, the data available to date allow us to consider ABCA1 and ABCG1 as important participants in the atheroprotection mechanism [279,280].

Caveolae and caveolin-1 act as an integral platform for reverse cholesterol transport involving ABCA1 [281,282]. Due to the molecular interaction in the plasma membrane and cytoplasm, caveolin-1 and ABCA1 are closely linked to cholesterol efflux through vesicular transport [283]. Indeed, in patients with Tangier’s disease and in Abca1^−/−^ mice, the transport of lipids mediated by caveolin-1 from the Golgi apparatus to the plasma membrane is defective [284].

The expression of ABCA1 and ABCG1 is regulated by liver X receptors (LXRs) [285,286,287]. It was found that native LDL increases the level of ABCA1 in endothelial cells at both the protein and mRNA levels, depending on the time and dose [288]. In contrast, oxLDL decreased ABCA1 levels in endothelial cells at both mRNA and protein levels in a dose-dependent manner by inhibiting LXRs [289].

Shear stress during laminar flow increases the expression of LXRs in endothelial cells through the PPARγ pathway, which is induced by laminar flow [290,291]. It was shown that the expression of LXRa, LXRβ and their target genes is higher in the endothelium of the mouse thoracic aorta, where laminar blood flow is assumed, than in areas with impaired flow, for example, in the aortic arch region [292]. Enhanced laminar blood flow production of LXR in mice increases the expression of Abca1 and Abcg1 in endothelial cells [292,293]. Thus, the laminar blood flow realizes its atheroprotective effect also through the reverse cholesterol transport, mediated by ABC transporters [292].

Interestingly, in experiments on overexpression of the ABCA1 gene in endothelial cells against the background of increased cholesterol outflow, no negative effect on key cellular functions was found [294]. Meanwhile, in lipopolysaccharide-stimulated endothelial cells overexpression of ABCA1 decreased markedly inflammation gene expression, confirming the information about the atheroprotective effect of the transporter.

## 6. Regulation of Lipid Permeability and Its Disorders in Atherosclerosis

The accumulation of lipids in the subendothelial space is one of the earliest observed stages in the natural history of atherosclerosis. Despite a long period of study, the mechanisms of this process are the subject of discussion. It is believed that LDL is the main atherogenic fraction that penetrates through the endothelial layer, accumulate and oxidise to form oxidized LDL (oxLDL) and are taken up by macrophages [295,296,297,298]. However, the way in which LDL penetrate into the subendothelial space remains incompletely understood.

Previously, this transit was explained by passive filtration. This mechanism explains well the increased lipid infiltration of intima in areas of arteries with turbulent blood flow, where endothelial cells do not have a polarized shape, undergo proliferation and have loose intercellular contacts. At the same time, intact endothelial cells provide dense intercellular connections with a width of about 3–6 nm, which allows only a limited number of substances, including some low-molecular-weight proteins, to pass through [299,300,301,302]. LDL are about 20–30 nm in diameter, which does not allow them to pass through the endothelial barrier freely [164,303,304,305]. In this regard, a theory was proposed about the presence of pores between adjacent cells in a continuous endothelial monolayer, which provided macromolecular transport [306,307,308,309,310]. Moreover, it was assumed that there were small and larger pores that could provide transport of large macromolecules [306,307,308]. However, this theory does not explain the entire transendothelial lipid transport [306].

Thus, the permeability of the barrier to LDL is provided not only by intercellular junctions, but also by other mechanisms. Studies have shown that the movement of LDL through the endothelial barrier is mainly due to transcytosis, which can be receptor-mediated or receptor-independent [302,306,311]. Transcytosis involves the transport of macromolecules using plasma membrane vesicles, which can be transported along with the substrate from the apical to the basal side of endothelial cells. These mechanisms include caveolae, scavenger receptors B1 (SR-B1), activin-like kinases 1 (ALK1) [164,197,312,313,314,315]. SR-B1 is an important participant in atherogenesis, as it mediates endothelial transcytosis of LDL and its subsequent accumulation in the arterial wall [316].

**Figure 3:**
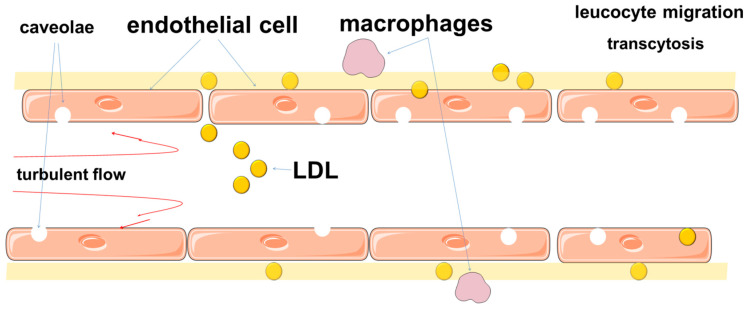
Schematic illustration of transcytosis involving caveolae.

Caveolae are thought to play a significant role in ensuring transcytosis (Figure 3) [303,317,318]. Endothelial transcytosis depends on the function of caveolin-1 [319]. These findings, together with data on the differential distribution of caveolae in endothelial cells from atheroprotective and atherosclerosis-prone regions of the aorta, confirm the role of Cav1 and caveolae as a central regulator of atherosclerosis [320].

Deletion of Cav-1 suppresses atherosclerosis by significantly attenuating LDL macromolecule transcytosis [164,197,315]. Interestingly, caveolin-1 deficiency impairs leukocyte adhesion to endothelium and extravasation into the arterial wall [191], through the effect of caveolin-1 on endothelial VCAM-1 expression as well as CCL-2 presentation and distribution at the leukocyte-endothelium interface.

The lipid composition of plasma membranes is also involved in the regulation of transcytosis [321]. Ceramide has been shown to increase endothelial permeability [321,322,323,324] and promote oxLDL transcytosis [221,303]. Ceramide production is increased by smoking [322,325,326,327], which induces endothelial cell barrier disruption in the lungs [322]. Tobacco smoke also disrupts endothelial cell monolayer integrity in a dose-dependent manner [322]. In experiments with pulmonary artery endothelial cells, ceramide has been shown to induce endothelial cell apoptosis and contribute to the weakening of intercellular connections throughout the monolayer [328]. In this regard, one item of information of interest is that smoking contributes to the formation of endothelial microparticles in the lungs, which are enriched with ceramides and may represent the main carrier of these sphingolipids in plasma. Acidic sphingomyelinase (aSMase), which induces ceramide production, is elevated in smokers and COPD patients not only in the lungs but also in blood plasma [329,330,331]. The significance of these effects is the subject of further discussions concerning the role of smoking and COPD in cardiovascular comorbidity.

It should be noted that endothelial transcytosis is not only associated with negative effects on atherogenesis, but may also provide atheroprotective effects. It is of interest to know that ABCA1 modulates apoA-I transcytosis through endothelial cells, which is part of an HDL-mediated mechanism of atheroprotection [332]. Moreover, HDL transport through endothelial cells is mediated by SR-BI and ABCG1 [333]. These data highlight the multifaceted role of endothelial transport in lipid homeostasis.

## 7. Endothelial Microparticles

Endothelial microparticles (EMPs) are a heterogeneous population of 100–1000 nm plasma membrane vesicles that are released into the bloodstream by endothelial cells during apoptosis and activation [334,335]. Moreover, EMPs associated with activation and apoptosis can have different phenotypes [40,336,337,338,339]. In normal plasma, EMPs are present in low concentrations, but they are significantly increased in atherosclerosis [340]. EMPs are considered as a marker of endothelial damage and the progression of atherosclerosis since they can reflect the balance between cell stimulation, proliferation, and apoptosis [335,340,341].

In addition to endothelial cells, microparticles released by smooth muscle cells, platelets, erythrocytes, and leukocytes were identified [341,342,343]. EMP of endothelial origin make up a smaller population of microparticles.

The diversity of EMP functions has yet to be evaluated, but it is already known that they can be involved in both physiological and pathological processes, influence angiogenesis activity, and are associated with the development of various CVDs, mainly initiated by endothelial dysfunction [341,344,345,346,347]. The action of EMPs is provided by the fact that they can act as a tool for intercellular information exchange by transferring many biological factors from the source cell [348,349]. By transferring some of their components to target cells, EMPs mediate cell activation, phenotypic modification and reprogramming of cell function. This makes it possible to consider EMPs as important regulators of the intercellular exchange of biological signals [350]. For example, a functionally active eNOS was identified in EMPs [351,352]. By delivering functional microRNAs to recipient cells, endothelial microparticles are involved in many functions of endothelial cells and other cells of the vascular wall [353,354,355,356]. EMPs are taken up by endothelial cells by receptor-mediated mechanisms or by phagocytosis [350,352,357]. Moreover, recipient cells more actively take up EMP-rich miRNAs, which is consistent with their communication function [356].

Hemodynamic characteristics of blood flow contribute to the regulation of EMPs release. Stable low shear stress in impaired blood flow increases endothelial apoptosis and stimulates EMP release through activation of Rho-kinases and ERK1/2 pathways [358]. A high level of shear stress, on the contrary, reduces the release of EMP as a result of endogenous release of NO and a subsequent decrease in ABCA1 expression [358]. ABCA1 is believed to play a role in the formation of microparticles during the outflow of cholesterol from cells [359]. Thus, the shear stress can be considered as a physiological regulator of EMP release. Interestingly, low physical activity in healthy individuals is also associated with increased levels of EMP circulating in the bloodstream, which may indicate the importance of hemodynamic characteristics of blood flow for endothelial function.

## 8. Conclusions

Endothelial cells are under the constant influence of physical factors related to blood flow patterns and blood pressure, which have different vectors. According to the current concept, shear stress is a key hemodynamic characteristic of blood flow. An important conceptual achievement has been the understanding that the endothelium is capable not only of detecting changes in blood flow patterns but also of responding adequately to these changes. Modifications in cell morphology and function ensure that the endothelium adapts to changing hemodynamic conditions.

Local changes in hemodynamics, along with the impact of risk factors, are early events in the natural history of atherosclerosis. This complex multifactorial process includes endothelial dysfunction as the main link. Recent studies have significantly improved our understanding of the processes associated with endothelial dysfunction, as well as the role of plasma membrane lipids in endothelial cell function.

It is assumed that the ability of endothelial cells to determine mechanical effects is associated with a complex of various factors. The endothelial mechanosome includes various structures, such as caveolae, as well as some molecules that provide the conversion of physical forces into molecular signals. Studies have shown a significant role of the biophysical characteristics of the plasma membrane of endothelial cells as a mechanism providing the reception and transduction of physical forces [183,360,361].

A comprehensive view of atherogenesis, including the analysis of not only biochemical, but also biophysical mechanisms, has expanded the boundaries of understanding the causes of atherosclerosis. Even in Virchow’s early works, the leading role of arterial wall damage followed by an inflammatory response was suggested [362]. This concept was extended in the works of Russell Ross, who proposed the ‘injury response’ hypothesis. This hypothesis allowed them to illuminate many aspects of atherogenesis from the position that atherosclerosis is not simply associated with mechanical lipid accumulation, but is the result of a more complex chronic inflammation-proliferative response to arterial endothelial damage [363,364].

At the same time, this analysis allows us to emphasize the importance of lipid metabolism as a central link in vascular biology. Indeed, current evidence suggests that the leading role of lipid metabolism in endothelial cell function is not only as a structural or energetic substrate, but also as a participant in cell mechanobiology. The evidence that lipids are at the intersection of chemo- and mechanobiological signaling pathways suggests a much more multifaceted role in atherogenesis.

This review demonstrates the complexity and versatility of the cross-linkages between hemodynamic characteristics of blood flow and lipid biology of the endothelium and opens prospects for possible further studies. They may be aimed at studying the physical characteristics of blood flow in areas with physiological turbulence, analyzing endothelial adaptation mechanisms to impaired blood flow, searching for tools contributing to the normalization of endothelial lipid homeostasis. A promising area for further research in this regard may be the study of ω-3 polyunsaturated fatty acids (PUFA) effect on the biophysical properties of endothelial plasma membranes, given their role in reducing cholesterol content in plasma membranes and influence on lipid raft structure [365]. The data on the differential effect on key biophysical characteristics of plasma membranes of different ω-3 PUFAs are of interest. The available data indicate that the chain length or unsaturation of ω-3 PUFAs is associated with different patterns of influence on the structural organization and dynamics of membrane lipids [366]. For example, eicosapentaenoic acid and docosahexaenoic acid have different effects on membrane bilayer width, fluidity, and cholesterol domain formation [366]. However, docosahexaenoic acid has a greater effect on increasing endothelial cell plasma membrane fluidity than eicosapentaenoic acid [367]. This may be due to the greater ability of docosahexaenoic acid to increase the unsaturation index in plasma membranes, as well as to its greater effect on reducing membrane cholesterol content or on the cholesterol/phospholipid ratio [367]. The incorporation of ω-3 PUFA into lipid raft phospholipids has great potential to modify their organized molecular architecture and to remodel lipid-protein interactions and, consequently, signaling pathways [365].

Thus, lipid metabolism plays an important role in endothelial cells and is a complex system, the keys to understanding all the disorders of which are still inaccessible to clinicians and researchers.

## Data Availability

Not applicable.
